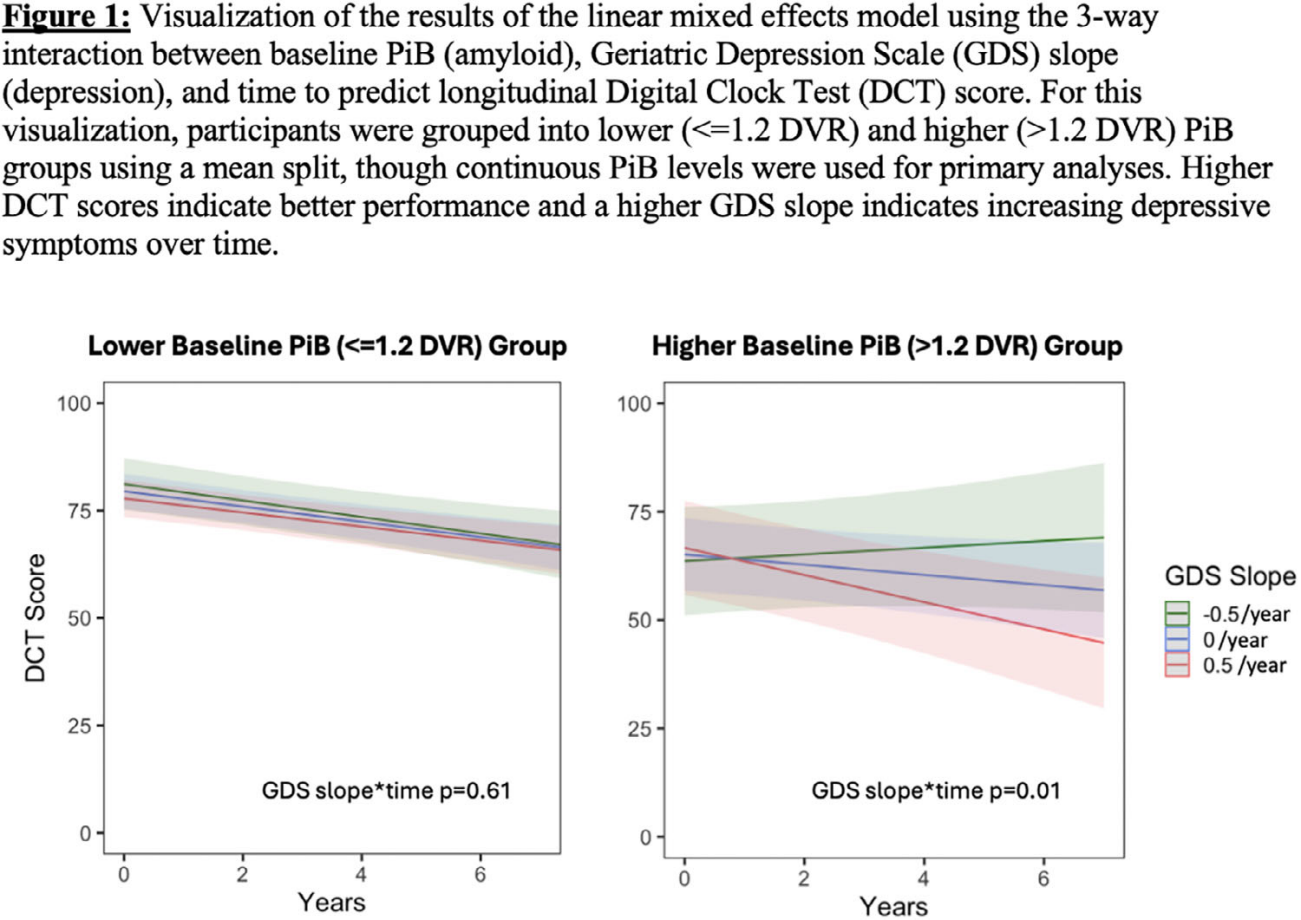# Impact of baseline amyloid and depressive symptom trajectory on longitudinal digital clock scores in community dwelling older adults

**DOI:** 10.1002/alz70857_104690

**Published:** 2025-12-25

**Authors:** Catherine E Munro, Abigail LaCasse, Jessie Fanglu Fu, Michelle E. Farrell, Talia L. Robinson, Natalie R Scher, Dorene M. Rentz, Bernard J Hanseeuw, Rachel F. Buckley, Michael J. Properzi, Patrizia Vannini, Rebecca E. Amariglio, Yakeel T. Quiroz, Deborah Blacker, Reisa A. Sperling, Keith A. Johnson, Gad A. Marshall, Jennifer R. Gatchel

**Affiliations:** ^1^ Harvard Medical School, Boston, MA, USA; ^2^ Brigham and Women's Hospital/Massachusetts General Hospital, Boston, MA, USA; ^3^ William James College, Newton, MA, USA; ^4^ Athinoula A Martinos Center for Biomedical Imaging, Massachusetts General Hospital, Harvard Medical School, Charlestown, MA, USA; ^5^ Massachusetts General Hospital, Harvard Medical School, Boston, MA, USA; ^6^ Athinoula A. Martinos Center for Biomedical Imaging, Massachusetts General Hospital, Harvard Medical School, Charlestown, MA, USA; ^7^ Brigham and Women's Hospital, Boston, MA, USA; ^8^ Massachusetts General Hospital, Boston, MA, USA; ^9^ Center for Alzheimer Research and Treatment, Department of Neurology, Brigham and Women's Hospital, Boston, MA, USA; ^10^ Institute of Neuroscience, Université Catholique de Louvain, Brussels, Belgium; ^11^ Department of Neurology, Massachusetts General Hospital, Harvard Medical School, Boston, MA, USA; ^12^ Brigham and Women's Hospital, Harvard Medical School, Boston, MA, USA; ^13^ Center for Alzheimer's Research and Treatment, Department of Neurology, Brigham and Women's Hospital, Harvard Medical School, Boston, MA, USA; ^14^ Department of Psychological & Brain Sciences, Boston University, Boston, MA, USA; ^15^ Grupo de Neurociencias de Antioquia, University of Antioquia, Colombia, Medellín, Antioquia, Colombia; ^16^ Harvard University, Boston, MA, USA; ^17^ Department of Epidemiology, Harvard T. H. Chan School of Public Health, Boston, MA, USA; ^18^ Department of Radiology, Division of Molecular Imaging and Nuclear Medicine, Massachusetts General Hospital, Boston, MA, USA; ^19^ Athinoula A. Martinos Center for Biomedical Imaging, Charlestown, MA, USA; ^20^ Department of Psychiatry, Massachusetts General Hospital, Harvard Medical School, Boston, MA, USA; ^21^ McLean Hospital, Belmont, MA, USA

## Abstract

**Background:**

The digital clock drawing test (DCT) is a computerized measure examining executive functioning, information processing, and visuospatial abilities. DCT scores have been shown to discriminate well between mild cognitive impairment and Alzheimer's disease (AD) dementia and have been associated with preclinical AD pathology (i.e., amyloid, tau). Prior work has shown that amyloid moderates the relationship between depression and performance on a standard cognitive composite score. However, it is unclear whether longitudinal trajectories of depressive symptoms impact DCT scores and whether amyloid levels influence such relationships. We sought to determine whether greater depressive symptoms were associated with a decline in DCT scores and whether baseline amyloid moderated this relationship.

**Method:**

Participants (*n* = 90, mean age=77.5±5.3; 58% female, mean education = 16.4 years) were cognitively normal (CN) and enrolled in the Harvard Aging Brain Study. The DCT (higher score=better performance) and Geriatric Depression Scale (GDS; higher score=greater depressive symptoms) were completed at baseline and annually thereafter (mean years follow‐up=5.7±0.9). Ordinary least squares regression slopes were calculated for GDS data. PiB‐PET (amyloid) was completed within 18 months of baseline assessments and examined using a large cortical aggregate. Separate linear mixed‐effects models assessed whether ‘GDS slope*time’ or ‘GDS slope*PiB DVR*time’ predicted longitudinal DCT scores, using continuous PiB values, and controlling for age, sex, education, and random intercept/slope.

**Result:**

GDS slope*time was not a significant predictor of longitudinal DCT scores. The 3‐way‐interaction with PiB was significant, such that those with greater PiB burden at baseline and higher GDS slopes showed declining DCT performance over time (beta=‐8.78,95%CI[‐14.66,‐2.90], *p* = 0.004) (Figure 1).

**Conclusion:**

In a cohort of CN older adults, individuals with higher baseline cortical amyloid burden and worsening depressive symptoms showed steeper declines on the DCT. These results suggest that individuals with greater/worsening depressive symptoms in the context of elevated cortical amyloid burden may be particularly vulnerable to cognitive decline (even in preclinical stages) on sensitive, easy‐to‐administer measures such as the DCT, similar to prior work examining cognitive testing composite scores. Findings highlight the clinical importance of monitoring emerging mood disturbances in older adults. Future work will examine how these relationships relate to clinical progression.